# Evaluating the feasibility and acceptability of a co-designed physical activity intervention for rural middle schoolers: a pilot study

**DOI:** 10.1186/s12889-024-19356-2

**Published:** 2024-07-09

**Authors:** Janette M. Watkins, Julia E. Brunnemer, Kathleen N. Heeter, Andrew M. Medellin, William C. Churchill, Janelle M. Goss, James M. Hobson, Nicole E. Werner, R. Glenn Weaver, Vanessa M. Martinez Kercher, Kyle A. Kercher

**Affiliations:** 1 Department of Kinesiology, School of Public Health-Bloomington, Indiana University, Bloomington, IN USA; 2 Program in Neuroscience, College of Arts and Sciences, Indiana University, Bloomington, IN USA; 3 Department of Health & Wellness Design, School of Public Health-Bloomington, Indiana University, Bloomington, IN USA; 4 Department of Applied Health Science, School of Public Health-Bloomington, Indiana University, Bloomington, IN USA; 5 Department of Epidemiology & Biostatistics, School of Public Health-Bloomington, Indiana University, Bloomington, IN USA; 6 Logan University, Chesterfield, MO USA; 7 White River Valley Middle School, Lyons, IN USA; 8 Department of Exercise Science, Arnold School of Public Health, University of South Carolina, Columbia, SC USA

**Keywords:** Youth, Sport-based youth development, Cardiovascular disease, Multilevel intervention, Physical activity, Feasibility testing

## Abstract

**Background:**

Lack of physical activity is a concern for children across diverse backgrounds, particularly affecting those in rural areas who face distinct challenges compared to their urban counterparts. Community-derived interventions are needed that consider the unique context and additional physical activity barriers in under-resourced rural settings. Therefore, a prospective pre-post pilot/feasibility study of *Hoosier Sport* was conducted over 8-weeks with 6th and 7th grade children in a low-socioeconomic rural middle school setting. The primary objective of the present study was to assess trial- and intervention-related feasibility indicators; and the secondary objective was to collect preliminary assessment data for physical activity levels, fitness, psychological needs satisfaction, and knowledge of physical activity and nutrition among participating youth.

**Methods:**

This prospective 8-week pilot/feasibility study took place in the rural Midwestern United States where twenty-four middle school students participated in a mixed-methods pre-post intervention during physical education classes. The intervention included elements like sport-based youth development, individualized goal setting, physical activity monitoring, pedometer usage, and health education. Data were collected at baseline (T1) and post-intervention (T3), with intermediate measures during the intervention (T2). Qualitative data were integrated through semi-structured interviews. Analytical methods encompassed descriptive statistics, correlations, repeated measures ANOVA, and thematic analysis.

**Results:**

Key findings indicate robust feasibility, with intervention-related scores (FIM, AIM, and IAM) consistently surpassing the “good” threshold and 100% retention and recruitment success. Additionally, participants showed significant physical performance improvement, shifting from the 25th to the 50th percentile in the 6-minute walk test (*p* < 0.05). Autonomy and competence remained high, reflecting positive perceptions of program practicality. Nutrition knowledge, initially low, significantly improved at post-intervention (*p* < 0.01), highlighting the efficacy of targeted nutritional education in Hoosier Sport.

**Conclusions:**

This study pioneers a community-engaged model for physical activity intervention in under-resourced rural settings. Positive participant feedback, coupled with improvements in physical fitness and psychosocial factors, highlights the potential of the co-design approach. The findings offer valuable insights and a practical template for future community-based research, signaling the promising impact of such interventions on holistic well-being. This research lays the foundation for subsequent phases of the ORBIT model, emphasizing collaborative, community-driven approaches to address the complex issue of declining physical activity levels among adolescents.

## Introduction

Cardiovascular disease (CVD) is the leading cause of death in the United States (U.S.), disproportionately affecting individuals from rural areas and lower socioeconomic backgrounds [1, 2]. Despite this burden, various risk factors contributing to CVD can be altered, and regular participation in physical activity (PA) stands out as a modifiable behavior effective in reducing CVD risk. Research indicates that when children establish PA habits during childhood, they’re more likely to continue these habits into adulthood. However, only 1 out of 5 children in the U.S. engage in recommended PA levels [3]. As atherosclerosis can begin in childhood, preventive strategies are crucial for instilling lifelong PA-related behaviors [4–6].

Lack of PA is a concern for children across diverse backgrounds, particularly affecting those in rural areas who face distinct challenges compared to their urban counterparts [7–11]. Indeed, research cites that urban children are more physically active than children in rural communities [12, 13]. Reduced lower socioeconomic status (SES) leads to limited sport and physical activity facilities [11], fewer parental role models for PA [7], and overall diminished parental support for physical activity [8]. Additionally, transportation limitations hinder access to education and extracurricular activities [9, 10], creating health and developmental disparities among children from varying socioeconomic backgrounds. Moreover, lower health literacy and educational attainment contribute to CVD risk factors [14], emphasizing the need for policies addressing socioeconomic disparities in schools. Prioritizing health-related initiatives like incorporating PA breaks, funding programs, and providing essential services such as opportunities for PA, transportation, and leadership development are vital for children’s health and well-being [14]. Children from lower socioeconomic households often face social stigma, exclusion from school teams, lack of sports equipment, and financial hardships. Notably, interventions targeting PA behaviors have shown limited success in improving population health and influencing PA trends [15, 16]. This highlights the necessity for improved interventions, especially concerning the persistent challenges of rural PA participation.

College students can play a significant role in shaping children’s behavior by imparting knowledge, skills, and values crucial for their development [17, 18]. Children perceive young adults as more relatable and credible compared to older adults, leading to effective communication about PA through interpersonal relationships such as role modeling. This often leads to an increased likelihood of behavior change [19]. Moreover, employing trained college student mentors as primary facilitators in interventions can enhance cost-effectiveness and sustainability [20]. This approach ensures a continuous flow of new student mentors, thereby reducing overall staffing costs. Community stakeholders frequently express dissatisfaction with short-lived programs that offer limited long-term benefits and lack sustained efforts [21]. To address these concerns, an implementation plan including college student mentors and a long-term collaborative approach with a rural middle school community was implemented through a novel sport-based physical activity intervention called *Hoosier Sport*. The intervention was created using human-centered participatory co-design with small groups of adults and children from the target community. This 5-step process included sessions focused on (1) problem identification, (2) solution generation, (3) solution evaluation, (4) operationalization, and (5) evaluation to come up with a prototype intervention protocol.

Considering the higher burden of cardiovascular disease (CVD) in rural populations [22, 23], there’s a growing need for innovative multilevel interventions in under-resourced communities. A multilevel intervention approach can potentially address health disparities at individual, interpersonal, and community levels. The present study follows/uses the Obesity-Related Behavioral Intervention Trials (ORBIT) model [24], which guides intervention development and testing to establish an evidence-based research foundation, increasing the likelihood of implementing effective multilevel interventions. Aligning the current pilot/feasibility project within the ORBIT model houses the current project within an efficient system that conceptualizes the project as a crucial step directed toward future efficacy trials. Building on our previous work co-designing an intervention with children and adults from the small rural community in the Midwestern United States (ORBIT Phase Ia: Design) [25], the present study moved into ORBIT Phase IIa preliminary testing for the *Hoosier Sport* intervention. The school partner has a student enrollment of 155 students. The entire school district provides free breakfast and lunch for all students based on the district’s high poverty metrics.

A prospective pre-post pilot/feasibility study of *Hoosier Sport* was conducted over 8-weeks with 6th and 7th grade children in a low-socioeconomic rural middle school setting. The primary objective of the present study was to assess trial- and intervention-related feasibility indicators; and the secondary objective was to collect preliminary assessment data for PA levels, fitness, psychological needs satisfaction, and knowledge of PA and nutrition among participating youth. Our primary hypotheses posited that *Hoosier Sport* would be feasible as defined by “good” scores for multiple trial-related and intervention-related feasibility indicators (“good” scores defined for each indicator in the Methods section and Table 1). Our secondary hypotheses were that the research team would feasibly collect preliminary clinical outcomes (e.g., 6-minute walk test, physical literacy assessments) and each of those outcomes would demonstrate a positive preliminary signal for improvement.

**Table 1:** “Good” feasibility scores

Feasibility Indicator	“Good” Threshold
*FIM*	Median of 16/20
*AIM*	Median of 16/20
*IAM*	Median of 16/20
*Recruitment*	*n* > 20
*Participant Retention*	~ 90%

Note: FIM = Feasibility of Intervention Measure; AIM = Acceptability of Intervention Measure; IAM = Intervention Appropriateness Measure

## Methods and analysis

### Setting and sample

**Fig. 1:**
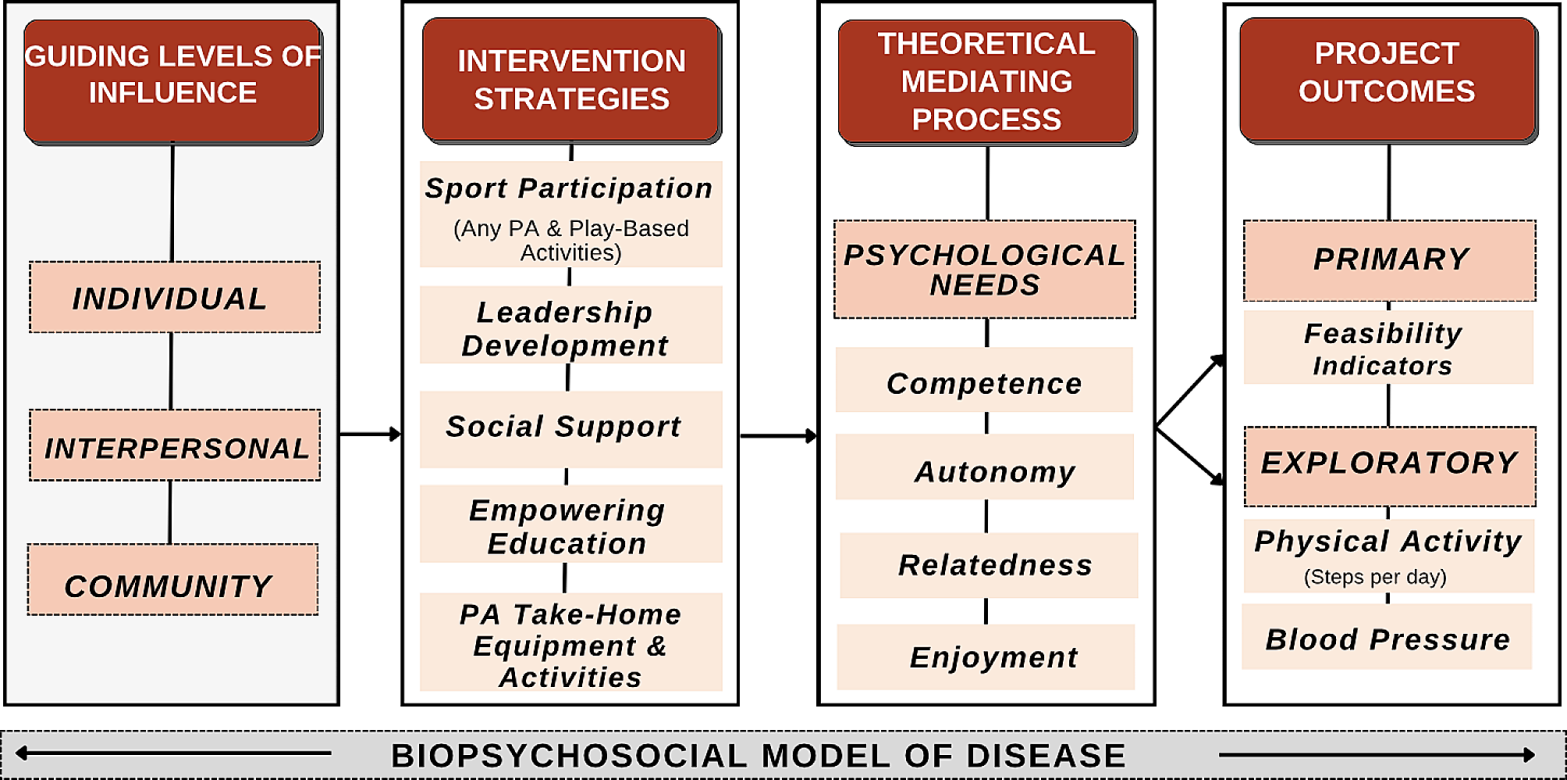
Hoosier sport conceptual framework

The sample for this study was comprised of children from a rural, low socioeconomic community in the Midwestern United States. Specifically, 6th and 7th grade students from a Midwestern middle school were recruited to participate in the study conducted mainly during physical education class. A total of 24 participants were included, comprising 58% females and 42% males, with ages ranging from 10 to 13 years (*M* = 11.92, *SD* = 0.98). Demographically, 94.4% of the participants identified as White, 2.7% as Black, and 2.7% as American Indian. All aspects of the research, including recruitment, intervention, assessments, and evaluations, took place within the school setting. The school setting provided a structured environment for the research team to interact with the participants. Figure 1 provides further detail of the conceptual framework of *Hoosier Sport.*

### Design

A Hybrid Type 3 design [26] was employed, enabling the examination of changes over time and the assessment of the Hoosier Sport intervention’s effects both at the individual and group levels. Hybrid Type 3 design allows for simultaneously assessing implementation feasibility indicators while evaluating information on clinical outcomes [26]. Furthermore, the Hybrid Type 3 design offers a robust framework that integrates qualitative and quantitative data, enabling a formative evaluation of both the intervention’s preliminary signals and the nuances of its implementation, ensuring a more holistic understanding of its impact [26].

### Intervention

**Fig. 2:**
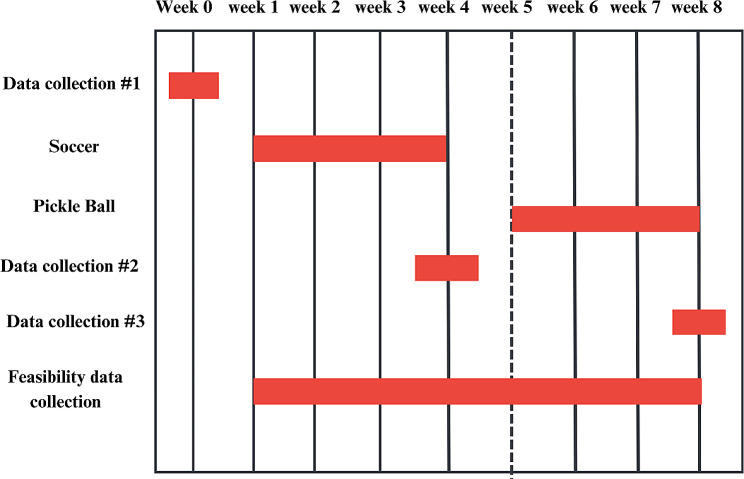
Timeline of data collection

The Hoosier Sport intervention spanned 8 weeks, organized into two 4-week sport units — soccer during weeks 1–4 and pickleball during weeks 5–8. The intervention targeted two levels-of-influence: individual (e.g., physical activity assessments, child’s perception of feasibility) and interpersonal (e.g., visual pedometer tracking board, class competition for pedometer wear, sport participation). The research team collected data at three key time points: week 0 (pre-intervention baseline assessment), week 4 (midway through the intervention), and week 8 (post-intervention evaluation), while also collecting feasibility data throughout (e.g., attendance, pedometer usage, step tracking). This systematic data collection approach was designed to provide insights into the participants’ experiences and changes in physical activity behaviors over the course of the study. See Fig. 2 for an overview of the intervention key time points.

### Measures

#### Trial-related feasibility indicators

To gauge the trial’s feasibility, several trial-related indicators were considered, including recruitment capability and retention. Recruitment capability assessed our ability to attract suitable participants; while retention, measured the participants’ willingness to participate through the duration of the study. Semi-structured interviews were conducted with high-attendance students to assess their motivation and overall sentiments towards *Hoosier Sport*. Selected from both 6th and 7th grades, three students in each grade were provided additional consent forms. Ultimately, two students from each grade returned the forms and participated in the semi-structured interviews, providing insights into their experiences with the program. A small group was chosen to assess the feasibility of conducting these interviews during the intervention (interviews occurred in week 7 of the intervention).

#### Intervention-related feasibility indicators

Intervention-related feasibility indicators were evaluated such as attendance, acceptability, appropriateness, and feasibility using 4-item measures adapted for children: AIM (Acceptability of Intervention Measure), IAM (Intervention Appropriateness Measure), and FIM (Feasibility of Intervention Measure), respectively [27, 28]. AIM, IAM, and FIM were assessed with a five-point Likert scale (“completely disagree” to “completely agree”). Summary scores for each measure were calculated with means/medians and standard deviations. The establishment of a “good” threshold was necessitated by the absence of standardized cut-off scores for AIM, IAM, and FIM. Higher scores are indicative of improved feasibility, acceptability, or appropriateness. Consequently, a score of 16 was chosen, representing the 75th percentile. Subsequent deliberations led the research team to designate 80% as a satisfactory threshold, aligning with the study objectives. Therefore, scores surpassing 16 were deemed as meeting the “good” criterion. Compliance was assessed through participants’ adherence to pedometer wear, goal setting, and step tracking, ensuring their active participation and adherence to the prescribed activities. A child-adapted version of the Basic Psychological Needs in Exercise Settings (BPNES) scale. was used to measure the fundamental psychological needs of autonomy, competence, and relatedness within the context of physical activity. The BPNES utilized a Likert scale ranging from 1 (indicating “complete disagreement”) to 5 (indicating “complete agreement”). The BPNES has been validated for use in children ranging from ages 5 to 16 [29].

#### Physical activity and health measures

Physical activity levels, fitness, and physical literacy were measured via the Canadian Assessment of Physical Literacy (CAPL-2) [30]. The CAPL-2 has demonstrated high reliability (α: 0.71–0.97) for participants under the age of 15 [31]. Specifically, self-reported moderate-to-vigorous activity (MVPA) was reported to measure physical activity levels (earning up to 5 possible points), knowledge was measured via the Knowledge and Understanding” subsection of the CAPL-2 (consisting of a potential 10-point scale) (e.g., “Cardiorespiratory fitness means:”). The cumulative correct answers were calculated as a percentage (e.g., 90%) to provide an overall measure for analysis. Scoring for the CAPL-2 was calculated using the corresponding test administration manual [32]. Similarly, nutrition knowledge was examined using questions covering MyPlate, with a possible 2 points, and a Likert scale of 1 to 4 (“strongly disagree” to “strongly agree”). Importantly, in addition to the Plank test, fitness was assessed with the 6-Minute Walk Test (6MWT). This test was chosen because it is accessible to all levels of fitness and has established normative values for various age groups and populations, aiding in the interpretation of results.

#### Environmental supports

This study utilized a Policy, Systems, and Environment (PSE) [33] questionnaire to assess environmental supports for physical activity and health. As a suitable questionnaire for PSE for children within the middle school context was unable to be identified, our PSE questions were compiled by a research team member with expertise in state government policy and rural health equity. Survey questions covered access to water within the school (e.g., water fountain), lunchtime fruit and vegetable choices, and exercise opportunities within the school. Additionally, the study explored the transfer of health-related knowledge to participants’ homes, gauging the dissemination and application of learned information about nutrition and physical activity beyond the school setting. This comprehensive approach aimed to provide valuable insights into the broader contextual support for healthy behaviors and the potential impact of educational interventions on participants and their families.

### Procedure

Children from a rural, low socioeconomic community were recruited through flyers distributed during school lunch hours and emails sent to parents. The inclusion criteria specified that students must be enrolled in 6th or 7th grade, actively participating in physical education (PE) during the semester of the intervention and deemed medically safe to engage in physical activity based on the Physical Activity Readiness Questionnaire (PAR-Q) [34] assessment. The criteria for inclusion were determined based on the Hoosier Sport taking place during PE classes, with the goal of boosting participation and attendance of the intervention. Children in 6t^h^-7th grade were selected because they are mandated to earn a PE credit at the community partner school. Interested children were contacted, and a parental call was scheduled to explain the study and obtain consent. Parents completed the PAR-Q [31]to assess their child’s eligibility via online survey. Children provided their assent during week 0 via an in-person survey with the research team, where they could choose to participate or not. This inclusion criteria and assent process was guided by the Indiana University Institutional Review Board to ensure the safety of the child participants. Upon agreement, participants received an identification code and proceeded to answer demographic and health-related questions, including inquiries about health literacy, nutrition knowledge, psychological needs, and questions addressing multi-level impacts such as family dynamics, school policies, and environmental factors.

All data collection and intervention delivery were conducted by college student mentors serving on the research team. After the assent process, participants underwent the 6-minute walk test (6MWT) [35]. Before the test, their blood pressure (BP) and heart rate (HR) were measured, and they rated their perceived exertion (RPE) before exertion on a scale of 1–10. The 6MWT was conducted with a college student researcher counting laps in the school gym. After the test, participants provided their RPE and had BP and HR measured again. Following a 5-minute break, participants performed the Plank test, after receiving instructions and a visual demonstration. After the Plank test, participants received a Hoosier Sport shirt and returned to class.

During the first day of the intervention (week 1, day 1), children set goals, including daily step goals, and received wrist-worn pedometers. They were instructed to wear the pedometers continuously, removing them only when showering or sleeping. At the midpoint of the intervention (week 4), qualitative data was collected from participants. This encompassed success stories, ongoing feedback regarding the intervention’s progress, and overall reflections on engaging in the program. Post-intervention data collection in week 8 replicated the procedures of week 0. Children answered survey questions, underwent health assessments, and shared success stories. A process evaluation was conducted to gather feedback from participants and the Hoosier Sport team for potential revisions. Pedometers were collected and feasibility assessments were conducted by tracking the percentage of days, recording attendance for the *Hoosier Sport* program, and monitoring the days the pedometers were worn. Participants were asked to record their daily step count but since pedometers were primarily used for feasibility, this data was not collected or used by the research team.

### Data analysis

Data was analyzed using the latest version of R Studio (2023.12.0 + 369), and figures were generated using the most recent version of MATLAB (R2022b). The exploration of pre- to post-intervention results encompassed key health measures, including HR, BP, Plank scores, and the 6-Minute Walk Test (6MWT). Paired t-tests were employed to assess the significance of within-subject differences between pre- and post-intervention measurements for each parameter. Specifically, the t-tests were conducted to compare HR and BP values, Plank scores, and distances covered during the 6MWT before and after the intervention. Finally, the qualitative analysis of data gathered from semi-structured interviews with children aimed to identify key aspects of the program that were enjoyable to them. This analysis involved a multi-step process to ensure the validity and reliability of the findings. First, two researchers independently coded the interview data, extracting themes and patterns that emerged from the children’s responses. These initial codes were derived from basic descriptive data, allowing for a systematic exploration of the children’s experiences and preferences within the program. Following the coding process, the researchers collaboratively organized all relevant quotes into these derived themes, providing a coherent and structured representation of the children’s perspectives. To further enhance the validity of the analysis, a qualitative expert conducted a thorough review of the data and themes. This expert review served as a validation step, ensuring that the interpretations and conclusions drawn from the data were grounded in robust qualitative analysis practices.

### Sample size

The primary goal of this pilot study was to assess the feasibility of the research methods, procedures, and interventions. Based on research guidelines, a sample of 20–30 is acceptable for pilot feasibility study [36, 37]. With 24 participants, we could comprehensively evaluate these aspects, identify logistical challenges, and refine protocols as necessary. Additionally, the sample size of 24 enables us to gather preliminary data that will be crucial for informing future research endeavors. By estimating effect sizes, understanding variability within the sample, and gaining initial insights into potential outcomes, we can make informed decisions about sample size calculations and study design for larger-scale studies.

## Results

### Trial-related feasibility indicators

In recruitment, there were 58 eligible students (in 6th or 7th grade, taking PE), comprising 33 from the 6th grade and 25 from the 7th grade. The objective was to enlist a minimum of 20 participants for the pilot study. In fact, we recruited 24 participants, 15 from the 6th grade and 9 from the 7th grade. All 24 participants were retained for the study’s duration.

**Fig. 3:**
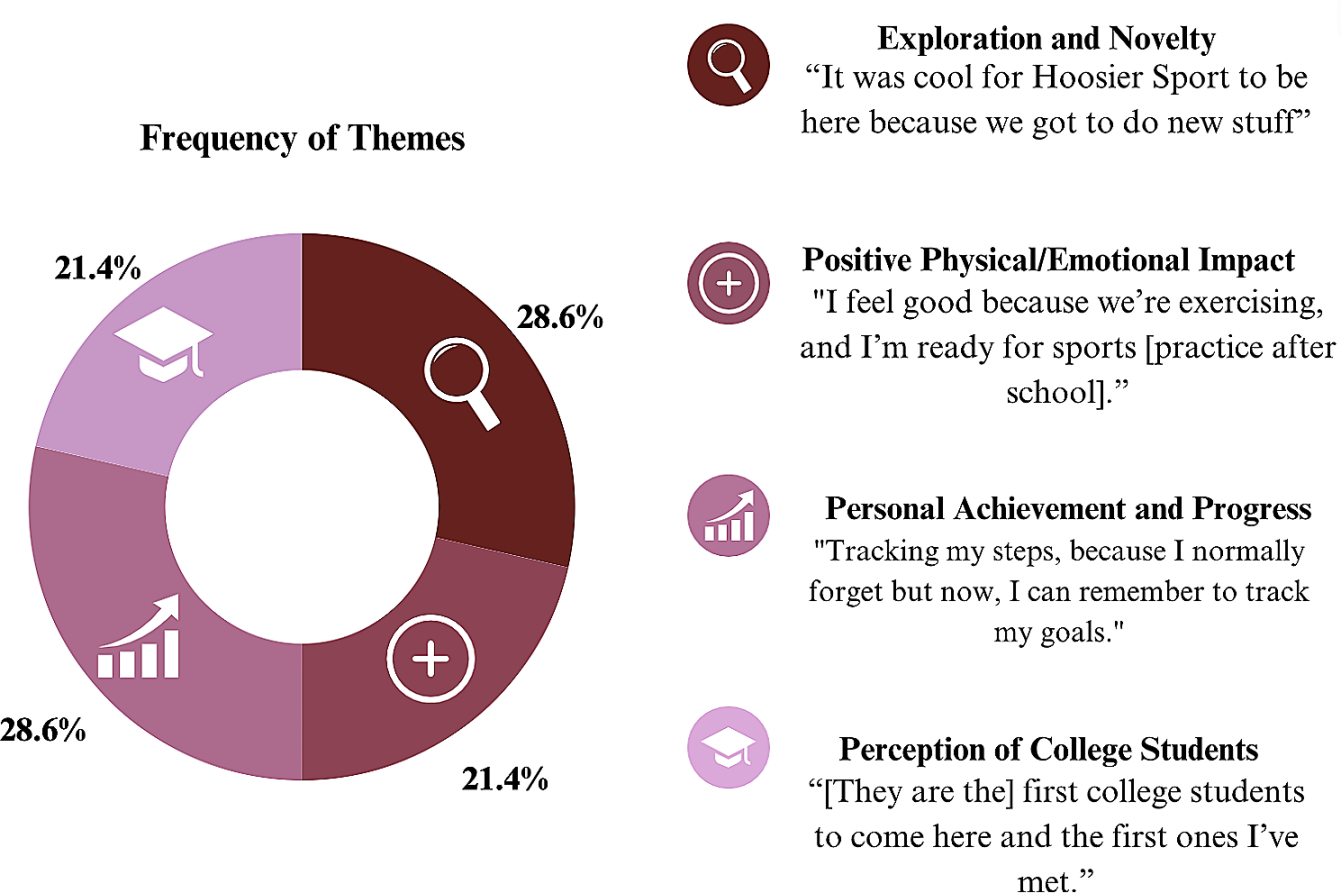
Themes in participant responses (*n* = 4)

Additionally, qualitative data was collected through semi-structured interviews with a select number of participants (*n* = 4). These students were asked five questions regarding their experience in Hoosier Sport. The most reported themes regarding their experience in Hoosier Sport included learning new skills in Hoosier Sport and achieving new goals. Figure 3 illustrates all the themes of children’s responses.

### Intervention-related feasibility indicators

A majority of participants, comprising over 91%, either agreed (*n* = 12) or completely agreed (*n* = 10) that Hoosier Sport “seemed likely to succeed.” Likewise, over 88% expressed agreement (*n* = 11) or complete agreement (*n* = 12) regarding the program’s feasibility, and over half of the participants (52%) fully agreed (*n* = 13) that the program was indeed doable. Concerning ease of participation, more than 61% of participants fully agreed (*n* = 16) that the program was easy to engage with, and over 57% provided complete agreement (*n* = 15) that Hoosier Sport met their approval. Similarly, over 88% either agreed (*n* = 11) or completely agreed (*n* = 12) that the program was exciting to them, while more than 57% fully agreed (*n* = 15) that they welcomed Hoosier Sport. Furthermore, over 69% of participants (*n* = 18) completely agreed that the program was appropriate, and approximately 73% provided complete agreement (*n* = 19) that Hoosier Sport was a good fit for their school. A substantial 84% of participants either agreed (*n* = 10) or completely agreed (*n* = 11) that *Hoosier Sport* was aligned with their interests, and 88% either agreed (*n* = 9) or completely agreed (*n* = 14) that the program was a good match for them. Mean score FIM was 19.5 *(SD* = 1.2), AIM was 18 *(SD =* 2.3), and IAM was 18 *(SD* = 1.1).

For the BPNES, scores of autonomy, competence, and relatedness were assessed. For autonomy, students at baseline reported that they “strongly agreed” they had autonomy in their exercise choices (*M* = 3.9, *SD* = 0.98). This high autonomy was mirrored at post-intervention (*M* = 3.68, *SD* = 1.22). Competence was also ranked high at baseline, with most students reporting they “strongly agreed” they were capable performing exercise/sports confidently (*M* = 4.66, *SD* = 3.47). This feeling of competence remained high at post-intervention (*M* = 4.7, *SD* = 4.03). Lastly, at baseline students reported that they “agreed a lot” regarding feeling related to their peers in exercise settings (*M* = 3.5, *SD* = 3.18). At post-intervention, there was a slight increase in this perception (*M* = 3.8, *SD* = 3.02). From pre- to post-intervention perceptions of autonomy (*p* = 0.871), competence (*p* = 0.443), and relatedness *(p* = 0.220) were not significantly different.

### Physical activity and health measures

**Fig. 4:**
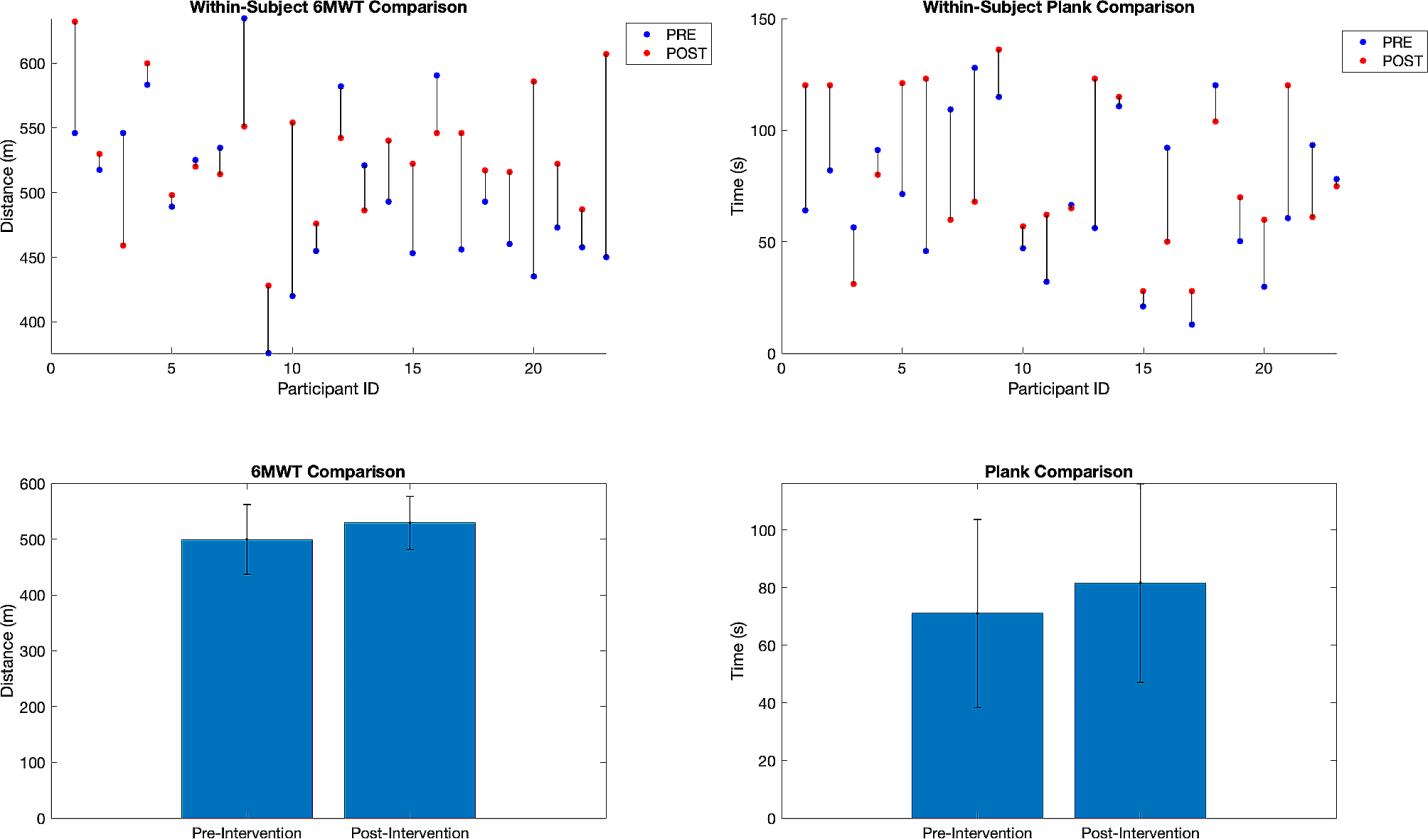
Pre- and post-intervention 6MWT and plank scores. The figure illustrates changes in the 6MWT and plank scores for individuals (top plots of figure) and at the group level (bottom plots of figure). Significance was seen in the 6MWT at the group level from pre- to post-intervention, but not for the plank score

Quantitative survey data was collected in person using an iPad, and physiological tests were conducted in the gymnasium. To explore potential changes in the 6MWT, a paired-sample t-test was conducted. This test found that the difference between pre- and post-intervention measurements was statistically significant (*p* = 0.026), with a mean difference of 31.03 m. However, the Plank test scores were not significantly different from pre- to post-intervention (*p* = 0.105), although there was an increase of 13 s on average. Figure 4 illustrates this data. For detailed statistical outcomes, please refer to Table 2.

**Fig. 5:**
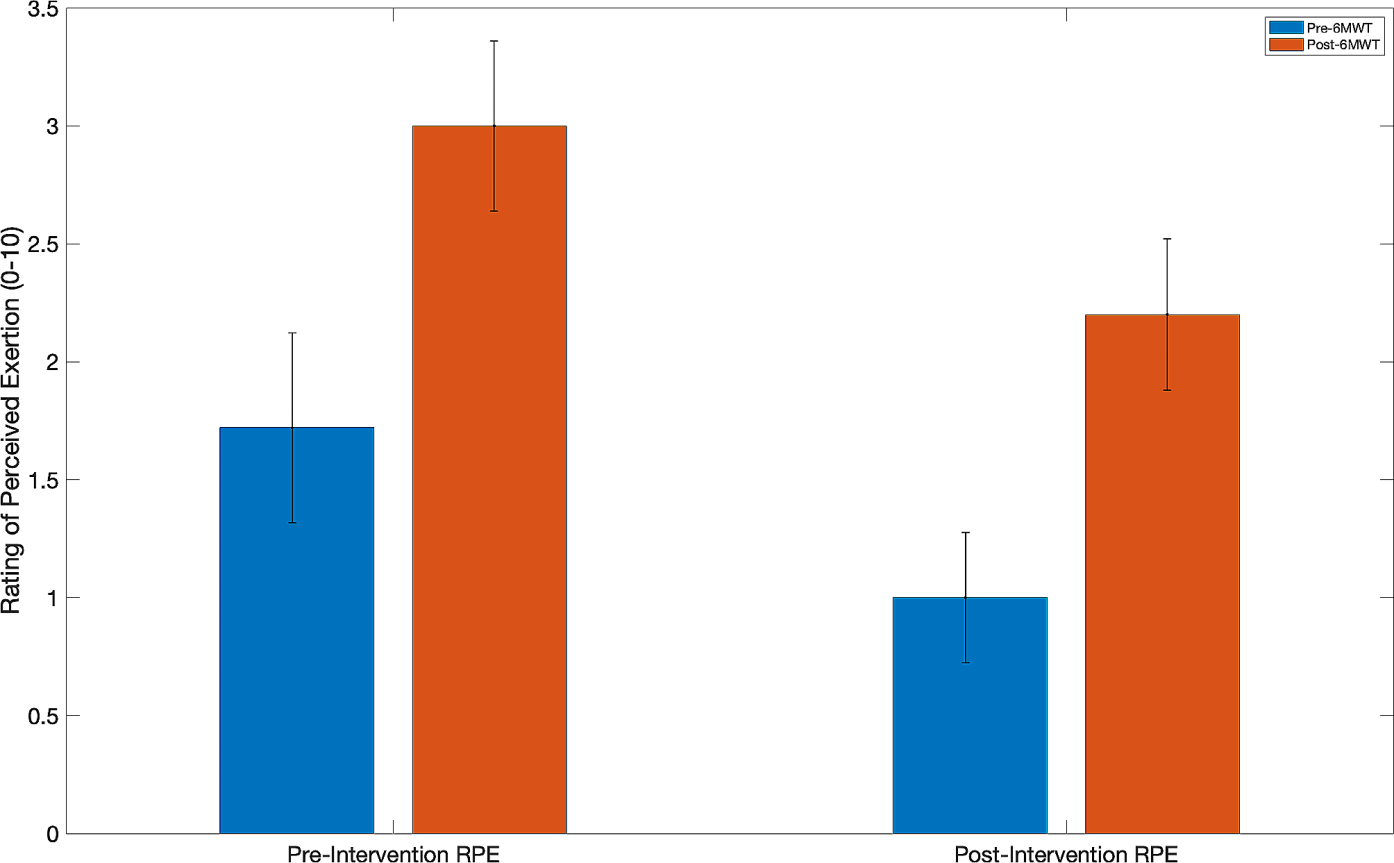
Mean RPE comparison pre- to post-6MWT and intervention. Changes in the rating of perceived exertion (RPE) from pre- (blue bars) to post-6MWT (red bars). Additionally, this change is represented over time from pre- to post-intervention (time points are noted on the x-axis). Pre- and post-6MWT RPE were significantly different for both the pre- and post-intervention time points

To examine changes in the RPE across the intervention, a paired-sample t-test was conducted. The analysis of RPE before the 6MWT revealed the difference between pre- and post-intervention measurements approached significance (*p* = 0.061). For post 6MWT measures, the changes were not significant (*p* = 0.069). Additionally, the analysis of RPE after the 6MWT yielded statistical significance at both pre-intervention (*p* = 0.003) and post-intervention (*p* = 0.003). Figure 5 illustrates these results by condition and time point. For detailed statistical outcomes, please refer to Table 2.

**Fig. 6:**
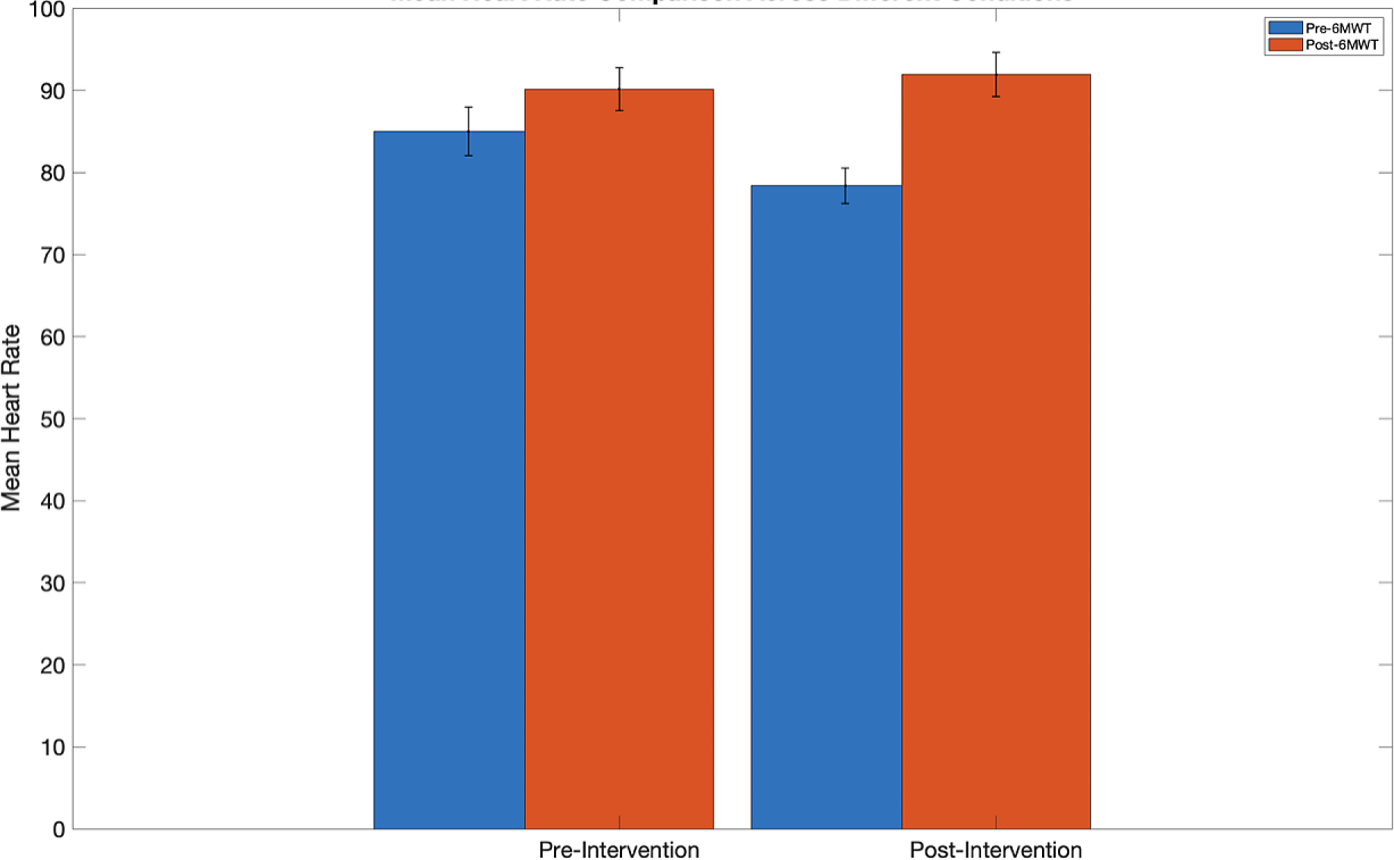
Mean heart rate comparison across 6MWT and intervention. This figure illustrates changes in the heartrate pre- (blue bars) and post-6MWT (red bars). Additionally, this change is represented over time from pre- to post-intervention (time points are noted on the x-axis). Pre- and post-6MWT heartrate were significantly different for both the pre- and post-intervention time points

The variations in HR before and after the 6MWT were assessed using a paired-sample t-test. The analysis indicated no significant difference in resting HR between pre- and post-intervention measurements (*p* > 0.05). Similarly, there was no significant difference in HR following the 6MWT from pre- to post-intervention (*p* > 0.05). However, significant changes in HR were observed before and after the 6MWT for both the pre-intervention and post-intervention periods. Figure 6 visually represents this data, illustrating the changes in HR over time for each condition. Please refer to Table 2 for all statistical details.

The study explored BP, examining systolic and diastolic pressure separately. For the pre-intervention time point, no notable differences were found in systolic pressure before versus after the 6MWT. Similarly, diastolic pressure showed no significant changes during this period. Conversely, at the post-intervention time point, there was a significant change in systolic pressure following the 6MWT, with a mean increase of 8.64 mmHg. Diastolic pressure, however, did not show a significant difference after the 6MWT. When considering the entire time span from pre- to post-intervention, resting diastolic and systolic pressure did not display significant differences. Nevertheless, a significant difference was observed between pre- and post-intervention systolic pressure, while diastolic pressure showed no significant change following the 6MWT. For all statistical results, please refer to Table 2.

**Table 2:** Paired T-tests results for physiological measures

	*Variable(s)*	T-Test			*Mean Difference*	Confidence Interval		*Cohen’s d*
		*t*	*df*	*p*		*Lower*	*Upper*	
Pre- vs. Post-Intervention	RPE Pre-6MWT	-1.959	23	0.061	-0.72	-0.038	1.478	-0.417
	RPE Post-6MWT	-0.979	23	0.337	-0.8	-1.491	0.531	-0.468
	HR Pre-6MWT	-1.575	23	0.128	-4.12	-1.276	9.516	-0.297
	HR Post-6MWT	1.146	23	0.263	3.84	-10.75	3.074	0.269
	S.BP Pre-6MWT	0.450	23	0.656	0.76	-2.724	4.244	0.074
	S.BP Post-6MWT	2.497	23	0.019	6.04	-11.031	1.048	0.475
	D.BP Pre-6MWT	0.1669	23	0.868	0.76	-10.161	8.641	0.056
	D.BP Post-6MWT	0.896	23	0.379	-2.8	-9.250	3.65	0.274
	Plank	1.686	23	0.104	13.2	-29.352	2.952	0.225
	6MWT	2.376	23	*0.025	31.032	4.083	57.980	0.527
Pre-Intervention	RPE Pre- vs. post-6MWT	3.216	23	*0.003	1.28	-0.458	2.101	0.670
	HR Pre- vs. post-6MWT	3.583	23	*0.001	5.76	-9.077	2.442	0.382
	S.BP Pre- vs. Post-6MWT	1.030	23	0.313	1.84	-5.525	1.845	0.169
	D.BP Pre- vs. Post-6MWT	0.439	23	0.664	1.28	-7.287	4.727	0.108
Post-Intervention	RPE Pre- vs. post-6MWT	3.2863	23	*0.003	1.2	-1.9536	0.4464	0.8
	HR Pre- vs. post-6MWT	5.3642	23	*< 0.001	13.72	-18.9988	8.4412	1.0688
	S.BP Pre- vs. Post-6MWT	5.2979	23	*< 0.001	8.64	-12.0059	5.2741	0.7103
	D.BP Pre- vs. Post-6MWT	1.1692	23	0.253	3.32	-9.1803	2.5403	0.2753

Note: RPE = Rating of Perceived Exertion, 6MWT = 6-Minute Walk Test, HR = Heart Rate, S.BP = Systolic Blood Pressure, D.BP = Diastolic Blood Pressure

The participants consistently demonstrated a high level of exercise knowledge throughout the intervention, as reflected in their CAPL-2 scores. At the baseline, students, on average, scored 64% on exercise knowledge questions (*SD* = 17%). Likewise, with post-intervention scores showing a slight decrease to an average of 61% (*SD* = 13%). A paired t-test showed no significant change in CAPL-2 scores (t (23)= -0.287, *p* = 0.774). In contrast, pre-intervention scores for nutrition knowledge were notably lower, averaging 13% (*SD* = 24%). However, there was a noteworthy improvement post-intervention, with an increase of 28%, resulting in an average score of 41% (*SD* = 15%) among students. This improvement in nutrition knowledge was significant from pre- to post-intervention (t(23) = 2.779, *p* = 0.008). For reported MVPA, at pre-intervention, participants reported an average of 4 days engaging in MVPA (*SD* = 1.85). At post-intervention, the average was 4.14 (*SD* = 1.68). This change was not significant between timepoints (t(23) = 0.642, *p* = 0.412).

### Environmental supports

PSE questions remained similar from pre- to post-intervention. At the end of the intervention, there was a modest increase in students receiving 5–10-minute physical activity breaks from teachers, shifting from “slightly true” (*M* = 2.07, *SD* = 0.85) to a slightly higher rating post-intervention (*M* = 2.11, *SD* = 1.05). Positive changes were observed in healthier food choices, with students moderately indicating their ability to choose vegetables or fruits during school meals (pre: *M* = 3.3, *SD* = 0.82; post: *M* = 3.5, *SD* = 0.78). Initial reports indicated that students found it moderately true that they could easily access water from school fountains when needed (pre: *M* = 3.23, *SD* = 0.8). However, post-intervention, there was a slight decrease in perceived ease of water access, falling to a moderate level (*M* = 3.01, *SD* = 0.88). The availability of evening use of school athletic facilities, although slightly reduced, still reflected moderate agreement (pre: M = 4.07, SD = 1.39; post: *M* = 3.86, *SD* = 1.46). Consistent behavior was observed in students moderately sharing physical activity information with their families (pre: *M* = 2.43, SD = 0.8; post: *M* = 2.46, *SD* = 0.73). Similarly, there was a slight increase in disseminating nutrition information post-intervention, reaching a moderate level (pre: *M* = 2.22, *SD* = 0.91; post: *M* = 2.29, *SD* = 0.84). The total change in PSE scores from pre- to post-intervention were not significant (t(23) = -0.169, *p* = 0.866). Overall, the findings suggest positive effects of the intervention on students’ physical activity and healthy eating choices, emphasizing the potential impact of targeted interventions in fostering health-conscious behaviors within a school setting.

## Discussion

This study aimed to assess the feasibility of a co-designed sport-based physical activity intervention delivered by college student mentors in 6th and 7th grade rural youths, while also collecting information on preliminary clinical outcomes. There were four key findings from this study. First, summary scores for intervention-related feasibility were high (FIM, AIM, and IAM all exceeding the “good” threshold), and trial-related feasibility measures exceeding targets with retention (100%) and recruitment (*n* > 20). Second, physical performance increased but perceived exertion decreased following the 8-week program finding. Third, autonomy and competence scores were high throughout the program. Lastly, nutrition knowledge was low at baseline, but scores increased at post-intervention. Each of these findings is discussed below.

The post-intervention assessment of *Hoosier Sport’s* acceptability, appropriateness, and feasibility provided valuable insights into participants’ positive perception of the intervention. An overwhelmingly positive response, with nearly all participants expressing confidence in the program’s success and endorsing its feasibility, indicates a high level of acceptance. Notably, participants unanimously endorsed the excitement and appropriateness of the intervention, reflecting alignment with their interests and school context. The favorable perception of *Hoosier Sport* extends to its practicality, emphasizing its real-world applicability. Certainly, the rationale behind adopting the Hybrid Design Type 3 approach lies in acknowledging that interventions demonstrating promise during initial stages of testing frequently produce less favorable outcomes in later testing phases. This trend is often attributed to the studies being conducted under suboptimal conditions, marked by insufficient support for adequate implementation, a lack of clarity regarding fidelity barriers, and the absence of implementation strategies aimed at overcoming those specific challenges [26]. A key factor contributing to this practicality is the seamless integration of the intervention into the under-resourced rural school setting. The ability for children to participate in *Hoosier Sport* during their regular PE class time played a pivotal role in supporting its practical nature. Two of the important considerations during the design of *Hoosier Sport* were leveraging and working within the existing school infrastructure (e.g., complex PE class schedules, student enrollment in PE, available gym space) and utilizing readily accessible college student mentors to deliver the intervention. This practical approach may have enhanced the participants’ perceptions of feasibility through increasing the likelihood that children could easily transition into and benefit from the program within the familiar school environment and from college students who are more relatable than regular adults.

The study outcomes unveiled consistently high scores across the AIM, IAM, and FIM among the majority of participating children, with each measure surpassing the predefined “good” threshold. These positive responses not only suggest a widespread perception of the *Hoosier Sport* intervention as acceptable and appropriate but also confirm its feasibility within the targeted population. The robust endorsement of these implementation outcomes, all exceeding the established “good” benchmark, aligns seamlessly with our initial hypothesis, providing compelling evidence that *Hoosier Sport* exhibits high feasibility. These favorable evaluations from the children underscore the potential success and seamless integration of the intervention into their context, providing valuable support for the viability of *Hoosier Sport* in practice. Furthermore, the strong acceptance among participants suggests the potential for long-term sustainability in participation [38]. Despite the challenges associated with conducting the intervention in a school setting, this approach and the accompanying reassuring feasibility results may be helpful as later stage intervention testing will also be conducted in similar community settings.

The results of the *Hoosier Sport* intervention revealed a significant improvement on participants’ physical performance and perceived exertion. The notable improvement in the 6MWT distances post-intervention suggests that the program may have contributed to enhancing cardiovascular fitness and endurance among participants [39]. Importantly, using national reference standards [40], the sample at pre-intervention were in the 25th percentile, and at post-intervention were in the 50th percentile. This outcome aligns with one of the intervention’s primary long-term goals of increasing overall physical activity levels, particularly evident in the observed increase in participants’ capacity to cover greater distances within the specified time frame. This finding is corroborated by existing research in sport-based youth development, which indicates that such programs contribute to positive health outcomes in children in under-resourced communities [41]. Similarly, the statistically significant alterations in HR during the 6MWT provide further insights into the intervention’s possible physiological benefits. These changes indicate potential cardiovascular adaptations, reflecting improved efficiency in handling the demands of sustained aerobic exercise [42]. This positive trend in fitness aligns with broader long-term outcomes targeted by the intervention, indicating a potential positive impact on participants’ functional capacity.

Throughout the intervention, participants consistently experienced high levels of autonomy and competence. Similar to other youth-based intervention studies, feelings of autonomy and competence were consistently strong, relatedness ranked comparatively lower [43, 44]. Conversely, the formative work for *Hoosier Sport* [45] revealed that relatedness was identified as the most important psychological need when formulating the intervention. Prior research has underscored the significance of addressing basic psychological needs in youth populations and highlights the potential long-term benefits it could confer on health [46]. Therefore, in line with our conceptual framework, future work within Hoosier Sport will continue to seek to increase these psychological needs.

In our examination of exercise and nutrition knowledge, pilot findings indicated a notable deficiency in the understanding of these topics among participants. Specifically, a pronounced gap exists in nutritional awareness, underscoring the urgent need for a more concentrated effort on educational sessions dedicated to nutrition. Importantly, our intervention exhibited a substantial improvement in nutritional knowledge from pre- to post-intervention, showcasing the efficacy of targeted educational approaches. Notably, *Hoosier Sport* incorporated a 20-minute nutritional segment comprising a brief lecture, worksheet, and small group discussions, which may have positively impacted participants’ nutritional knowledge. However, a parallel strategy was not employed for exercise knowledge, potentially explaining the observed stagnation in the CAPL-2 data. Future iterations of Hoosier Sport may benefit from a refined focus on educational strategies, such as integrating bi-weekly lectures and educational activities, to comprehensively enhance health and nutrition education in children. Indeed, prior research investigating nutrition curriculum has established that short-term interventions possess the potential to significantly enhance nutrition knowledge and promote healthier habits among children [47].

Despite the valuable insights gained from this study, certain limitations should be acknowledged. Firstly, the study’s focus on rural youths in 6th and 7th grades that were primarily White may limit the generalizability of findings to urban settings, other age groups, or children from other racial or ethnic backgrounds. Additionally, the study’s sample size, while consistent with the typical scale for a feasibility study [48], remains relatively small. This modest sample size could potentially constrain the generalizability of findings and hinder the statistical examination of effect size, as well as limit the scope of exploratory analyses [49].

The absence of a control group also hinders our ability to establish causation, as external factors could contribute to the observed changes. Indeed, the immediate next step for the research team is to conduct a trial with control comparators. Additionally, the lack of racial and ethnic diversity within the study sample limits the generalization of findings to more diverse populations. Furthermore, while the feasibility assessment of *Hoosier Sport* provides valuable insights, the study did not explore potential moderating factors that could influence the program’s acceptability and appropriateness. Additionally, while the feasibility and psychological needs measures (i.e., AIM, IAM, FIM, BPNES) have adequate psychometric properties in adult populations, within the present study they were adapted for children and therefore may not be as reliable and valid as the original measures. Acknowledging these limitations highlights the need for cautious interpretation of results and suggests directions for future research to address these constraints and further refine our understanding of interventions in youth populations.

## Data Availability

The datasets used and/or analyzed during the current study are available from the corresponding author on reasonable request.
